# Numerical simulation of laser-produced plasma expansion on a droplet surface

**DOI:** 10.1038/s41598-023-31069-x

**Published:** 2023-03-11

**Authors:** Zhenyu Zhao, Weizhong Li

**Affiliations:** Key Laboratory of Ocean Energy Utilization and Energy Conservation of Ministry of Education, Dalian University of Technology, Dalian, 116024 China

**Keywords:** Laser-produced plasmas, Fluid dynamics, Pure mathematics

## Abstract

In this study, a numerical model of the plasma expansion on a droplet surface based on the initial plasma method was proposed. The initial plasma was obtained through the pressure inlet boundary condition, and the effect of ambient pressure on the initial plasma and adiabatic expansion of the plasma on the droplet surface, including the effect on the velocity and temperature distribution, were investigated. The simulation results showed that the ambient pressure decreased, leading to an increase in the expansion rate and temperature, and therefore a larger plasma size was formed. Plasma expansion creates a backward driving force and eventually envelops the entire droplet, indicating a significant difference compared to planar targets.

## Introduction

Laser-produced plasma (LPP) have been widely studied in many applications, such as inertial confinement fusion, pulsed laser deposition in material science, and lithography^1,2^. Extreme ultraviolet (EUV) lithography is considered a promising technology for the production of next-generation semiconductor devices with resolutions below 5 nm^3,4^. LPP has been developed for the EUV light source because of its high efficiency, power scalability, and spatial freedom around plasma^5,6^. In the EUV light source, metal droplets as targets are irradiated by pulsed laser to create hot dense plasma and emit EUV light.

When a high-power laser irradiates the droplet surface, it will cause violent deformation and breakup of the droplet. Pulsed laser-induced propulsion and strong deformation on a water droplet were studied by Klein et al.^7^. The apparatus to control and visualize was discussed in detail^8^. Although the fluid-dynamics response of metal- and water-droplet by laser impact is analogous, the propulsion mechanism is remarkably different^9^. The main reason is the generation of high-temperature plasma on the surface of the metal droplet.

The evolution of plasma plays a key role in the process of generating EUV from metal droplets irradiated by a high-power laser. In particular, the parameters of the plasma state determine the absorption of laser energy and EUV radiation characteristics. Sato et al. measured the spatial profile of electron density, electron temperature, and average ionic charge by using a Thomson scattering (TS) technique^10^. They found that the spatial profile differed with different plasma conditions. Under all plasma conditions, intense EUV emission was only observed at a sufficiently high electron temperature and in an adequate electron density range. Sasaki et al.^11^ pointed out that high conversion efficiency (CE) is obtained with low-density plasma, which is produced by firstly irradiating a tin microdroplet by the pre-pulse laser to produce the preformed plasma. After the pre-pulse laser irradiation, the plasma expands up to 10 times the initial radius and results in the density decreasing to 0.001 the solid density. Schupp et al.^12^ found that laser intensity is the pertinent parameter setting the plasma temperature and the tin-ion charge-state distribution when varying laser pulse energy and duration, which would help to obtain high CE. High-energy ions in the plasma expansion would contaminate the optics. Understanding the plasma expansion dynamics would be beneficial for optimizing debris mitigation techniques^13–15^. In addition, plasma expansion also affects the droplet’s deformation, which has not been sufficiently understood. Therefore, the study of plasma expansion is of great significance to EUV light sources.

Numerical simulation is an effective method of exploring plasma physics in laser ablation processes. Laser-produced plasma expansion consists of two stages, isothermal expansion during the pulse and adiabatic expansion after the pulse^16^. For numerical simulations, it is necessary to model the plasma generation first, which mainly includes the interactions between laser and target and laser energy absorption in plasmas.

Moscicki and Hoffman^17,18^ have developed a theoretical model for the interaction of the laser beam with the target and the next with the evaporated material and investigated the effect of the laser wavelength on parameters of laser-ablated carbon plasma. Galasso et al.^19^ proposed a unified model for laser ablation silicon, which is used to determine the distribution of laser energy between the target and the plasma. Three fundamental mechanisms have been identified as the main factors: the transition from evaporative to volumetric mass removal occurring at the critical temperature, the collisional and radiative processes underlying the initial plasma formation stage, and the increased impact of the liquid ejection mechanism. Wang et al. further considered the plasma-shielding effect to simulate the pulsed laser ablation of an aluminum target using finite element analysis^20^.

From the review of numerical studies about plasma, it is evident that numerical models would be very complicated if plasma mechanisms were considered comprehensively and bring a great challenge for developing numerical models. However, the adiabatic expansion of the plasma is also critical for the EUV light source. In a double pulse shooting system, it affects the deformation of the droplet target after the pre-pulse and the emission of the EUV after the main pulse. The complexity of physical models during the pulsed laser has prevented researchers from conducting further studies on the adiabatic expansion of the plasma. Therefore it is considerable to develop a more efficient model to deal with these complex physical processes based on appropriate assumptions.

Su et al.^21^ developed a simplified radiation hydrodynamic model that considers the radiative transfer equation to investigate the radiation properties and dynamic evolution of highly charged ions in a laser-produced plasma in vacuum. Aggoune et al.^22^ studied the expansion characteristics of a metallic vapor assuming an initial plasma distribution. These works ignored the complex physical mechanism of plasma formation and treats the plasma with a certain shape and state as the initial distribution to study its adiabatic expansion process, which can make the physical model of plasma greatly simplified.

In the simplified models, since the shape and state of the initial plasma need to be assumed first, it is defined here as the initial plasma method. Although some physical mechanisms are ignored in the simplified models, valuable findings can still be obtained, proving that the initial plasma method is very effective^23^.

Thus far, the initial plasma method has been used only for planar targets, and there is no model suitable for droplet targets. Because of the spherical shape of the droplet, there exists no constraint on the expansion beyond the droplet diameter, and the plasma expansion on the droplet surface cannot be analyzed theoretically as in the case of a planar target^24^. The initial plasma method needs to be extended to accommodate more situations. The target surface is irritated by a laser pulse and ejects vapor particles due to phase change, which will be ionized into plasma^25^. The plasma continues absorbing laser energy and expands which can be identified as isothermal expansion until the laser pulse is terminated. Although the physical process is quite complex, it can be regarded as plasma dominated. For numerical modeling, it is reasonable to assume that the plasma ejects directly from the target surface in a certain state. Thus the physical model is simplified in terms of high-energy physics and will be more efficient in fluid dynamics. Under this assumption, the initial plasma method can be utilized to simulate plasma expansion, plasma propulsion, and plasma-induced droplet deformation.

In this paper, a numerical model of plasma expansion on the droplet surface based on the initial plasma method is proposed. The initial plasma was obtained through the pressure inlet boundary condition. The effect of the ambient pressure on the initial plasma and the adiabatic expansion of the plasma on the droplet surface, including the effect on the velocity and temperature distribution, are investigated.

## Mathematical modeling

### The governing equations

To reduce the complexity of the problem, assumptions are made for simplicity. The plasma is in local thermodynamic equilibrium (LTE) and is considered as a mixture of two species: metal vapor and ambient gas. The flow is treated as a compressible fluid and follows the ideal gas law.

The governing equations are the conservation of mass (continuity), Eq. (1), momentum, Eq. (2), and energy, Eq. (3).

1 $$\frac{\partial \rho }{{\partial t}} + \nabla \cdot \left( {\rho {\mathbf{U}}} \right) = 0{,}$$

2 $$\frac{{\partial \left( {\rho {\mathbf{U}}} \right)}}{\partial t} + \nabla \cdot \left( {\rho {\mathbf{UU}}} \right) = - \nabla p + \nabla \cdot \left[ {\mu \left( {\nabla {\mathbf{U}} + \nabla {\mathbf{U}}^{T} } \right)} \right]{,}$$

3 $$\rho c_{p} \left( {\frac{\partial \left( T \right)}{{\partial t}} + \left( {{\mathbf{U}} \cdot \nabla } \right)T} \right) = \nabla \cdot \left( {\kappa \nabla T} \right) - {4}\pi \varepsilon_{{\text{r}}} {,}$$

where *ρ* is the density, U is the velocity vector, *p* is the pressure, *μ* is the dynamic viscosity, *C*_*p*_ is the specific heat, *κ* is the thermal conductivity and *ε*_*r*_ is the net emission coefficient (NEC).

The transport equations for the standard *k-ε* turbulence model are:

4 $$\frac{\partial }{\partial t}\left( {\rho k} \right) + \frac{\partial }{{\partial x_{i} }}\left( {\rho ku_{i} } \right) = \frac{\partial }{{\partial x_{j} }}\left[ {\left( {\mu + \frac{{\mu_{t} }}{{\sigma_{k} }}} \right)\frac{\partial k}{{\partial x_{j} }}} \right] + G_{k} - \rho \varepsilon - Y_{M} {,}$$

5 $$\frac{\partial }{\partial t}\left( {\rho \varepsilon } \right) + \frac{\partial }{{\partial x_{i} }}\left( {\rho \varepsilon u_{i} } \right) = \frac{\partial }{{\partial x_{j} }}\left[ {\left( {\mu + \frac{{\mu_{t} }}{{\sigma_{\varepsilon } }}} \right)\frac{\partial \varepsilon }{{\partial x_{j} }}} \right] + C_{1\varepsilon } G_{k} \frac{\varepsilon }{k} - C_{2\varepsilon } \rho \frac{{\varepsilon^{2} }}{k}{,}$$

where *k* is the turbulence kinetic energy, *ε* is its dissipation rate, *μ*_*t*_ is the turbulent viscosity, *Y*_*i*_ is the mass fraction of each species, *G*_*k*_ represents the generation of turbulence kinetic energy, *Y*_*m*_ represents the contribution of the fluctuating dilatation in compressible turbulence, *C*_*1ε*_, *C*_*2ε*_, *σ*_*k*_, and *σ*_*ε*_, are constants.

The species transport equations in turbulent flows are:

6 $$\frac{{\partial \left( {\rho Y_{i} } \right)}}{\partial t} + \nabla \cdot \left( {\rho {\mathbf{U}}Y_{i} } \right) = \left( {\rho D_{i}^{m} + \frac{{\mu_{t} }}{{Sc_{t} }}} \right)\nabla Y_{i} {.}$$

In the transport equation, *Y*_*i*_ is the mass fraction, *Dm i* is the mass diffusion coefficient (m^2^/s), and *Sc*_*t*_ is the turbulent Schmidt number.

### Computational domain and boundary conditions

**Figure 1:**
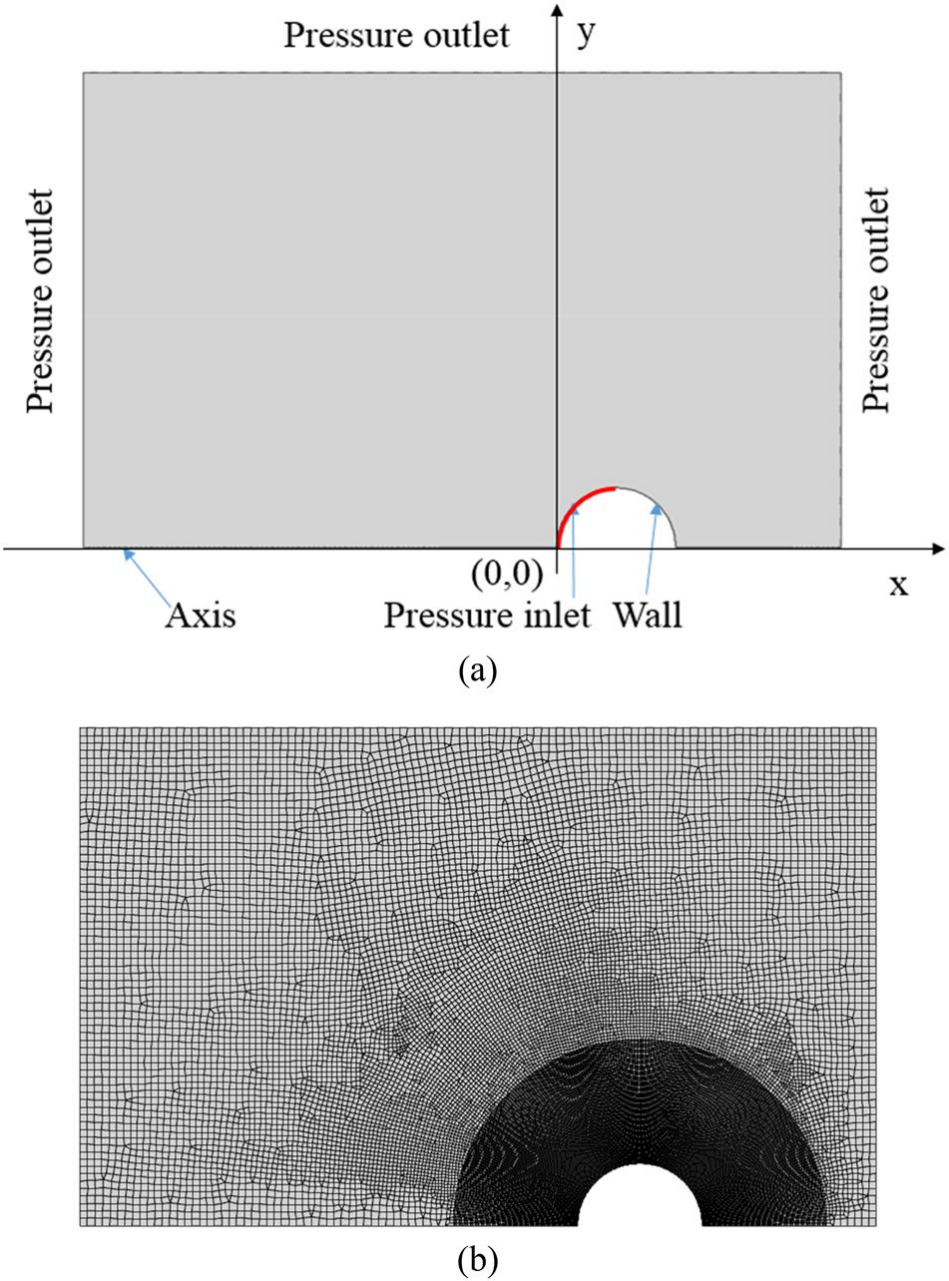
Schematic of (**a**) computational domain, and (**b**) meshing.

The computational domain is axisymmetric with a dimension of 1 mm × 0.6 mm as depicted in Fig. 1a. The semicircle represents the droplet surface and the droplet radius *R*_*0*_ = 25 μm. The left semicircle where the laser illuminates and initial plasma originates is designed as an entrance of material injection (marked as the front side) and the right semicircle is treated as a wall (marked as the back side). The physical model will be described in detail below.

Because the plasma expands at a tremendous rate, compressibility must be considered. To simulate the process of plasma ejection from the front surface, the pressure inlet boundary condition is used. The initial plasma is formed due to laser irradiation, with typical pulse durations ranging from 3 to 10 ns^9,26–29^, so here the pressure inlet is kept for 3 ns to obtain the initial plasma distribution, after which no more material is ejected, and the boundary condition is set as a wall. The initial plasma temperature and number density for the pressure inlet boundary condition are *T* = 30,000 K and *n*_*a*_ = 1 × 10^19^ cm^−3^, which are taken from typical plasma thermodynamic parameters^30,31^. Because the flow field is in a supersonic state at this stage, a mesh refinement of the region 200 µm outside the droplet surface is required to obtain more accurate simulation results and to maintain the stability of the numerical iterations, see Fig. 1(b).

Since Kurilovich et al.^32^ pointed out that the plasma has a noticeable effect on the droplet target within 30–50 ns after the end of the laser, the adiabatic expansion of the initial plasma was simulated for 30 ns and the effect of ambient pressure on the initial plasma as well as the adiabatic expansion were investigated. The governing equations are solved numerically in Ansys Fluent 16.0.

### Transport properties of plasma

**Figure 2:**
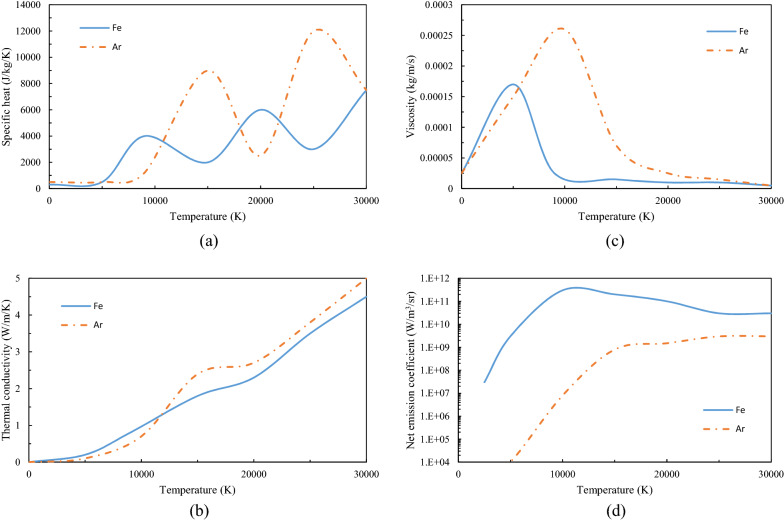
Transport coefficients of the plasma (**a**) specific heat, (**b**) thermal conductivity, (**c**) viscosity, and (**d**) net emission coefficient.

Here the plasma is assumed to be a mixture of iron vapor and argon, and the transport coefficients are taken from Murphy's calculations^33^. In hot plasma, the radiation transport cannot be ignored, and effective treatment is the net emission coefficient (NEC) method^34,35^. The transport properties of the plasma are plotted in Fig. 2 as a function of temperature.

## Results and discussions

### Plasma expansion verification

**Figure 3:**
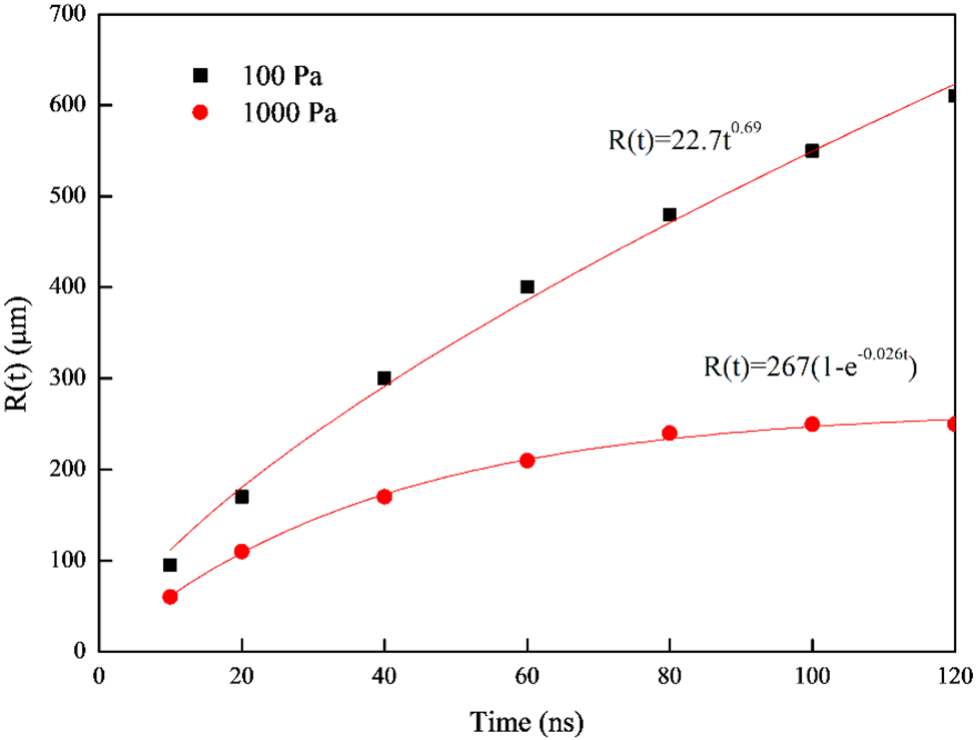
Plasma expansion distance as a function of time at different ambient pressures.

To verify that the model can simulate plasma expansion, plume dynamics need to be investigated quantitatively. After the initial plasma has been formed, the plasma expansion distance* R*(t) (with respect to (0,0) along the x-axis) at ambient pressures of 100 Pa and 1000 Pa are compared in Fig. 3. It can be seen that it follows the shock model at an ambient pressure 100 Pa and follows the drag model at an ambient pressure of 1000 Pa. The simulation results are in good agreement with the experimental study of Sharma et al.^36^. It is worth noting that the expansion distance as a function of time in vacuum follows a linear relation, which is different from that in ambient gases^37,38^.

**Figure 4:**
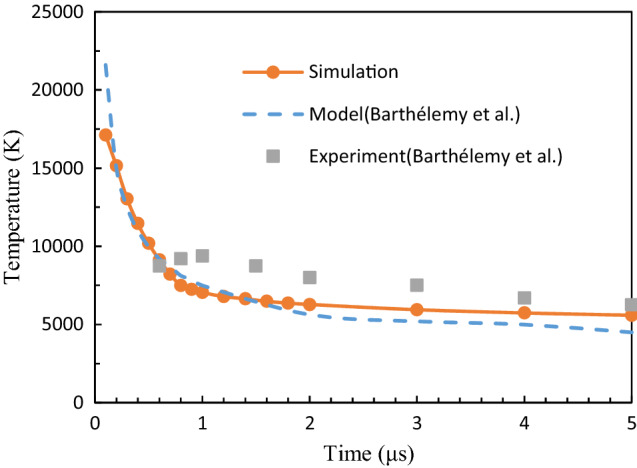
Time evolution of the plasma temperature obtained from the (**a**) simulations, (**b**) model calculations, and (**c**) experimental measurements by Barthélemy et al.^39^.

Temperature is a key parameter of the plasma. For further verification of the temperature distribution from the model, the simulations are performed with a target size of 0.4 mm^2^ and a laser pulse duration of 6 ns at atmospheric pressure, which accommodates the experimental setup by Barthélemy et al.^39^ The plasma temperature obtained from the simulation is compared with data from measurements and model calculations as depicted in Fig. 4. It can be seen that the temperature of the plasma drop substantially until 1 μs, after which it progressively slows down. The simulation results are in good agreement with the experimental and calculation data.

### Effect of ambient pressure on initial plasma

**Figure 5:**
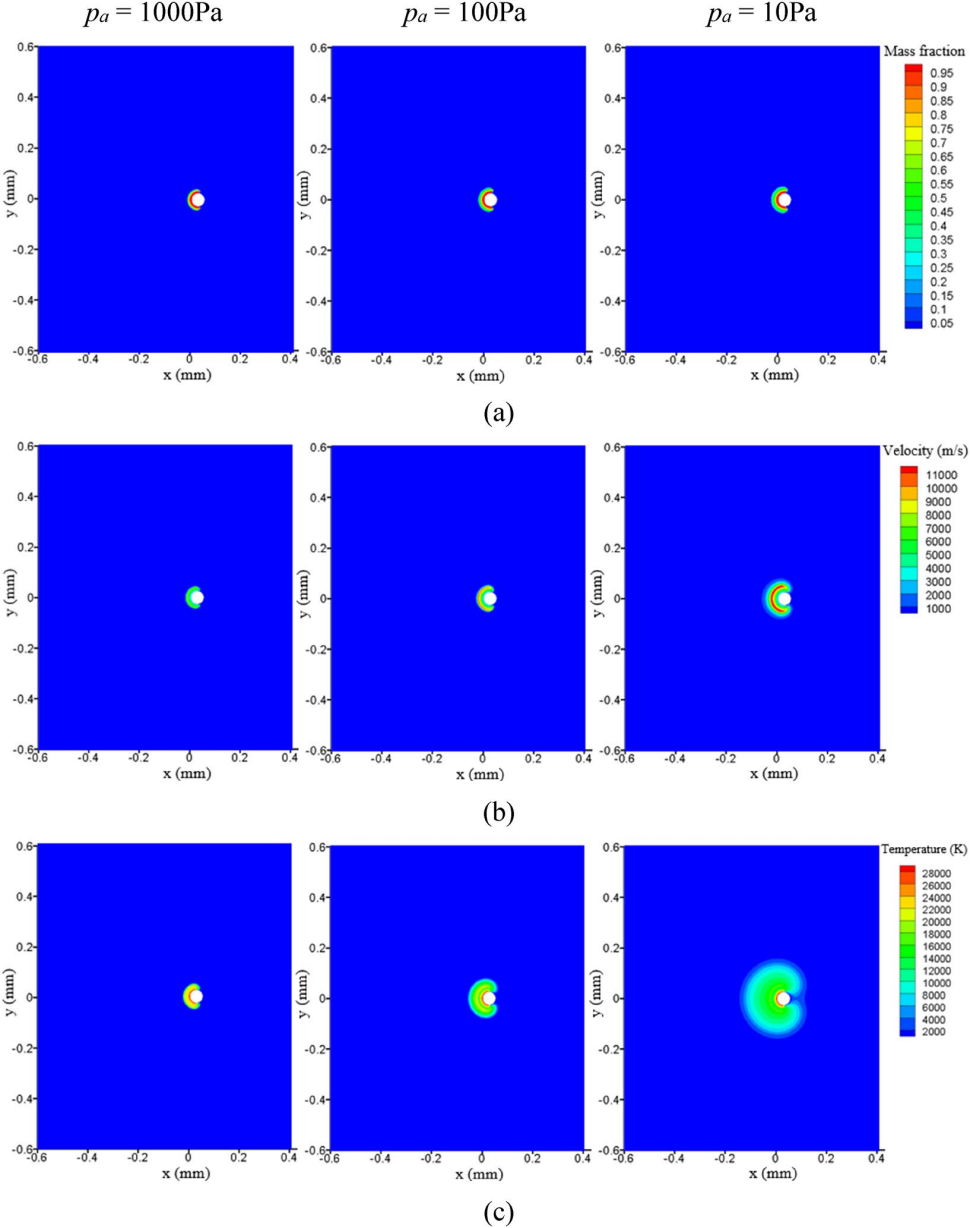
Effect of ambient pressure on (**a**) mass fraction, (**b**) velocity, and (**c**) temperature for the initial plasma.

The mass fraction, velocity, and temperature distribution of the initial plasma on the droplet surface at different ambient pressures are shown in Fig. 5, where *p*_*a*_ is the ambient pressure. The initial plasma size is larger at a relatively lower ambient pressure. The presence of shock fronts at the outer edge is manifested as a region of maximal values in the velocity and temperature contours, as shown in Fig. 5b,c. This model can predict the generation of shock waves during the rapid expansion of the plasma, which has been widely observed in experiments when a laser hit a target^40,41^.

**Figure 6:**
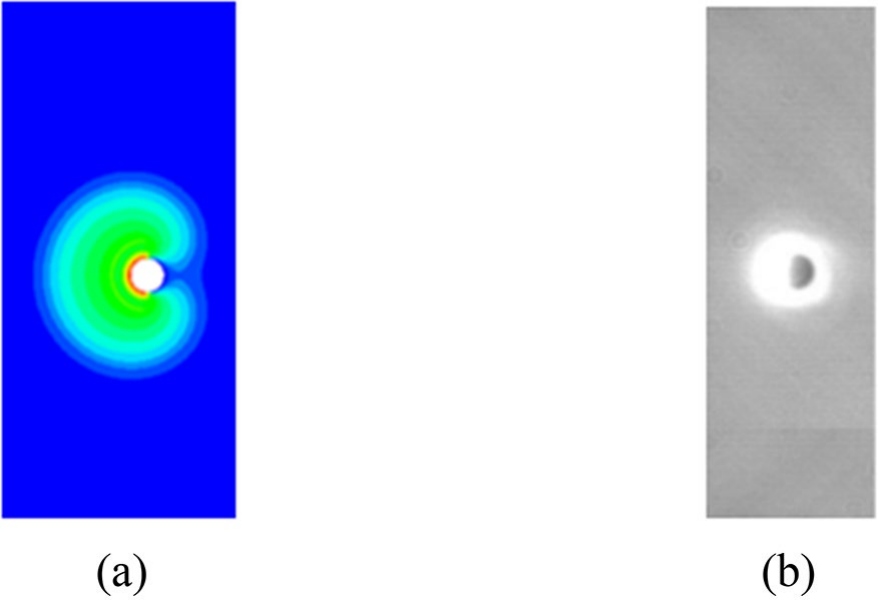
(**a**) The initial plasma, and (**b**) image of laser-produced tin droplet plasma in the experiment by Kurilovich et al.^9^.

**Figure 7:**
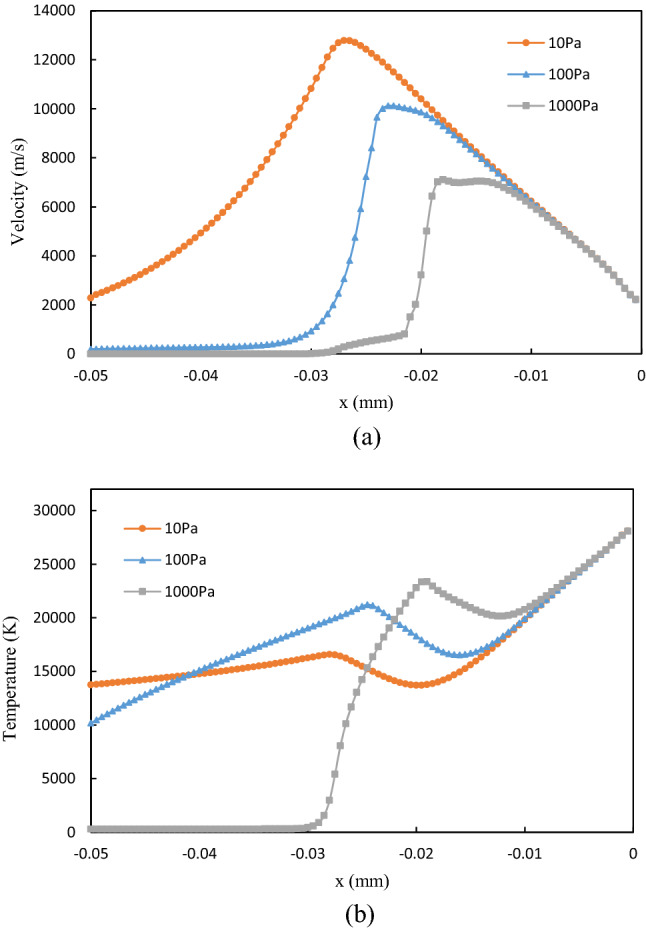
(**a**) Velocity, and (**b**) temperature profiles at the axis in the initial plasma.

As the plasma is imaged due to its high temperature emitted light, the temperature distribution of the initial plasma is compared with the image captured at the very beginning of the laser-produced tin droplet plasma experiment^9^, and the results are depicted in Fig. 6. It can be seen that at the early stage of plasma formation on a droplet surface, the droplet target has been immersed within the hot plasma. The simulation results are consistent with the experimental observations. The velocity and temperature profiles on the axis are shown in Fig. 7. It can be seen that at low ambient pressure the plasma can obtain a greater expansion velocity, which implies a larger dimension of the initial plasma. The temperature reaches an extreme value at the position of the velocity maximum, which is due to the jumps in physical parameters such as velocity, temperature, pressure, and density that occur before and after the shock front^42,43^.

### Effect of ambient pressure on plasma expansion

**Figure 8:**
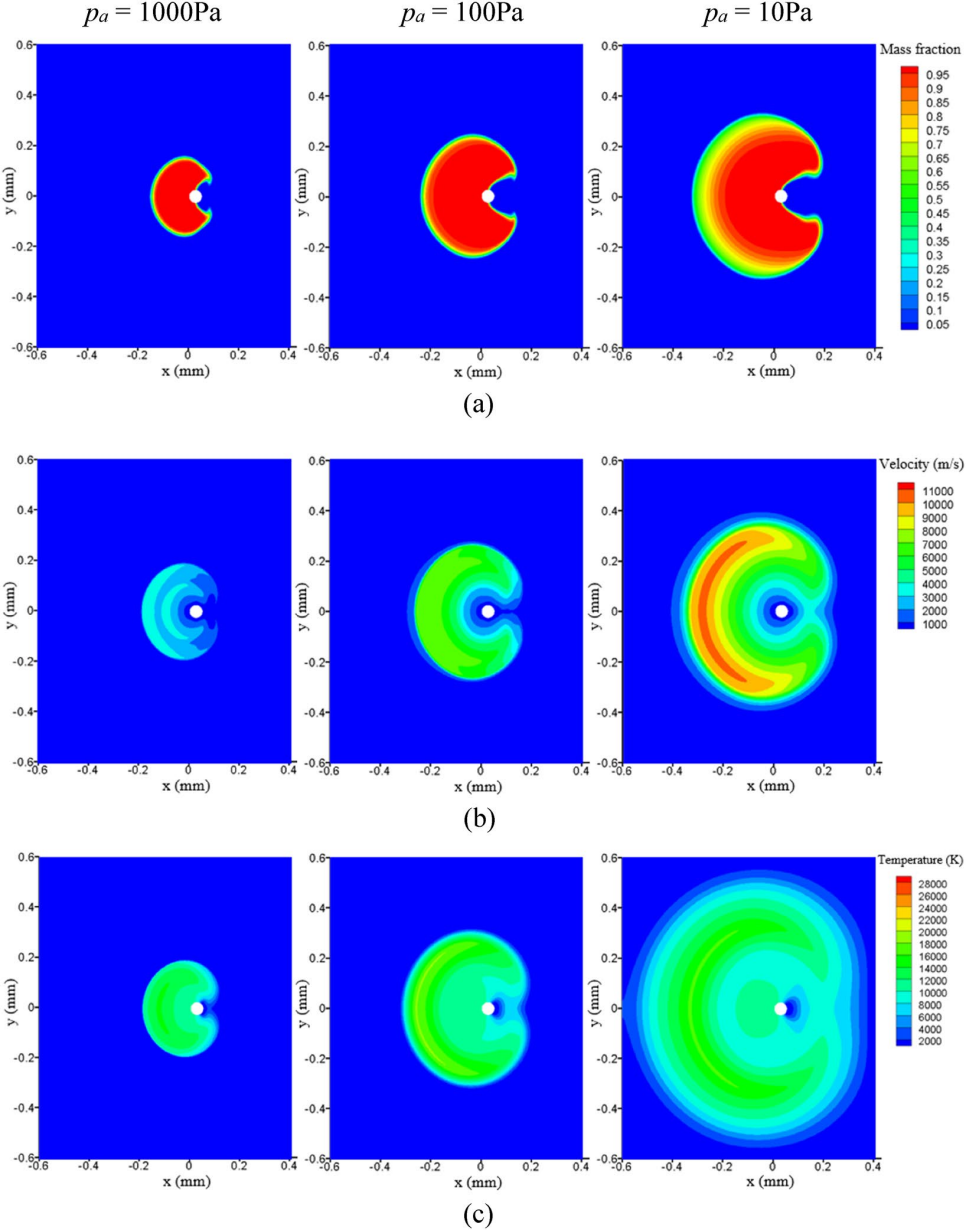
Effect of ambient pressure on (**a**) mass fraction, (**b**) velocity, and (**c**) temperature for plasma expansion at 30 ns.

The effect of ambient pressure on the adiabatic expansion of the plasma on the droplet surface is shown in Fig. 8. The plasma expands both to the front and to the back of the droplet, tending to encircle the droplet, and the tendency to encircle is more remarkable at lower ambient pressures. This phenomenon is significantly different from the plasma expansion on a planar target because the droplet does not confine the plasma expansion.

**Figure 9:**
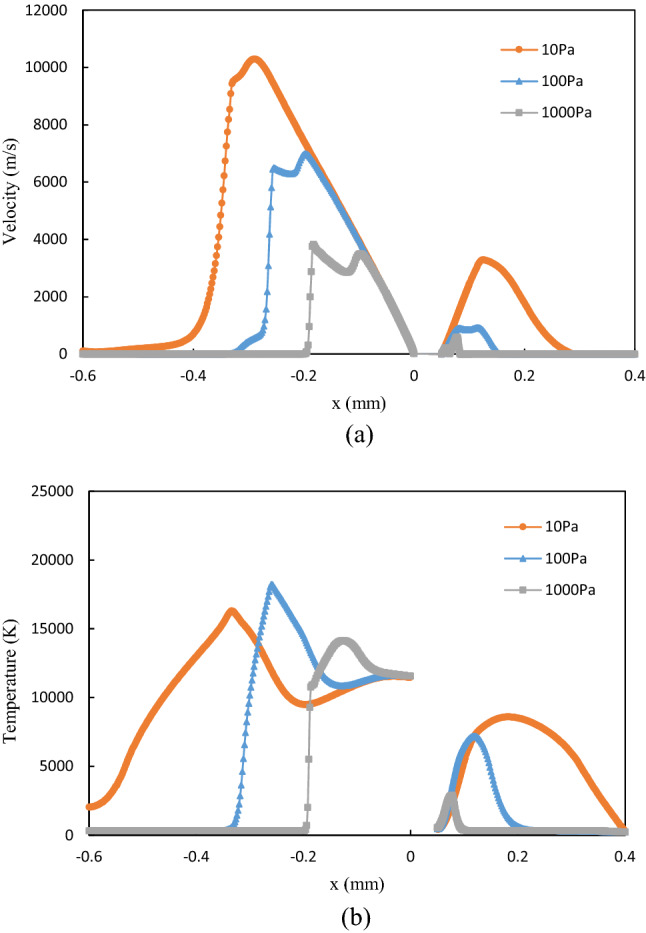
(**a**) Velocity, and (**b**) temperature profiles at the axis for plasma expansion at 30 ns.

The velocity and temperature profiles on the axis at 30 ns are plotted in Fig. 9. Since the ambient gas weakens the kinetic energy of the plasma expansion, the increase in ambient pressure leads to a significant decrease in the maximum values of velocity during the adiabatic expansion of the plasma. Meanwhile, the plasma loses more energy due to heat conduction and radiation at a higher ambient pressure, resulting in a lower temperature.

**Figure 10:**
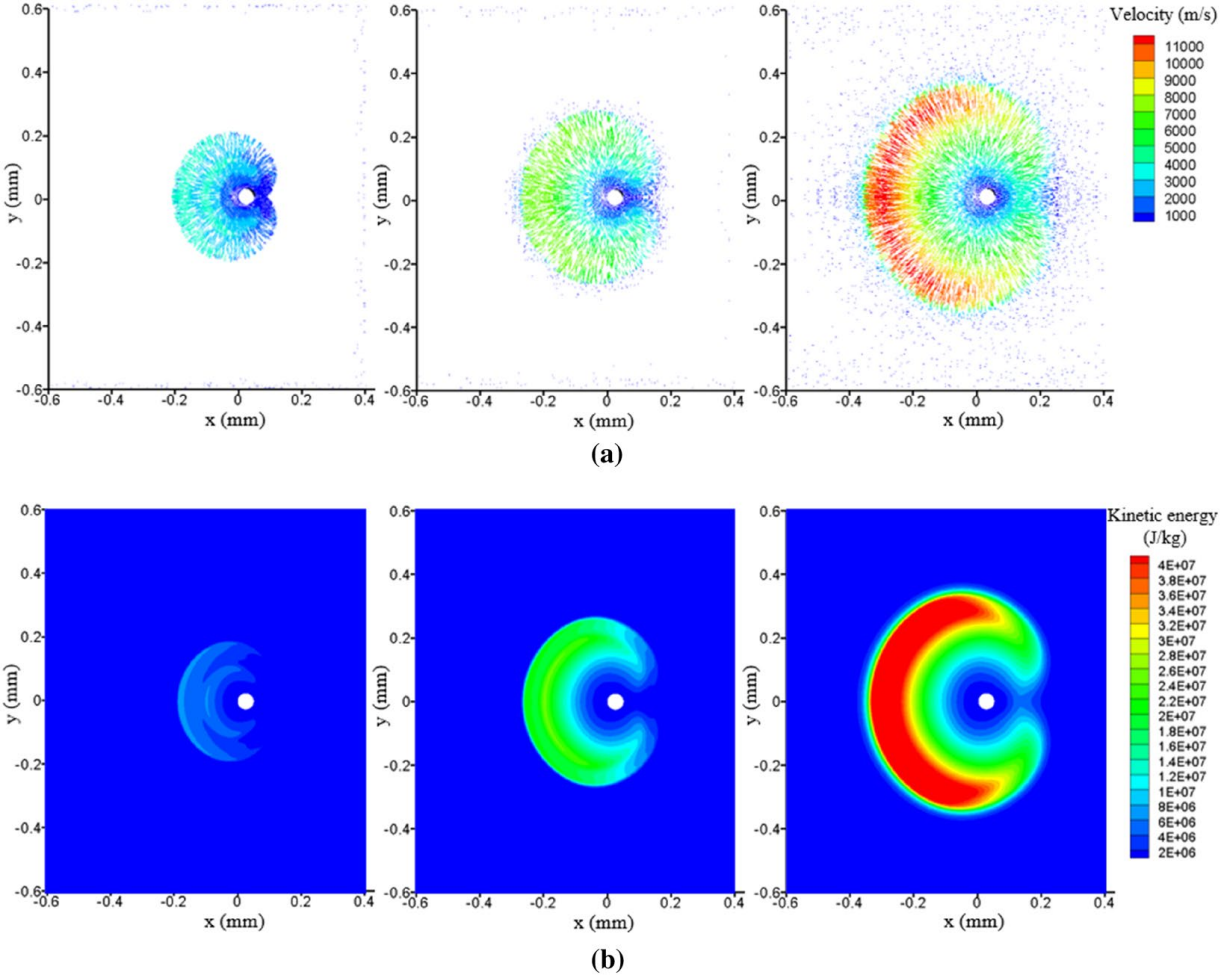
Effect of ambient pressure on plasma expansion (**a**) velocity vectors, and (**b**) kinetic energy.

Figure 10 illustrates the velocity vector and kinetic energy distribution in the plasma expansion. It can be found that the plasma expands radially with the droplet as the center from Fig. 10a. The lower the ambient pressure, the greater the kinetic energy of expansion as well as the impact on the backside of the droplet.

## Conclusions

A numerical model of plasma expansion on the droplet surface based on the initial plasma method is proposed in this paper. The effects of ambient pressure on the initial plasma and the adiabatic expansion of the plasma on the droplet surface are investigated. In general, the ambient pressure decreases, leading to an increase in expansion rate and temperature and therefore the formation of a larger plasma size. The plasma on the droplet surface expands radially and affects the back of the droplet as well, indicating that a backward driving force is generated. Eventually, the plasma will envelop the entire droplet target, which is a significant difference from the plasma expansion on a planar target. Based on this model, the ion distributions as well as the spectral properties of the EUV light source can be further investigated if ionization and radiative transfer are taken into account.

## Data Availability

The datasets generated during and/or analysed during the current study are available from the corresponding author on reasonable request.

## References

[1] Scheers J (2020). Time- and space-resolved optical stark spectroscopy in the afterglow of laser-produced tin-droplet plasma. Phys. Rev. E..

[2] Giovannini AZ, Gambino N, Rollinger B, Abhari RS (2015). Angular ion species distribution in droplet-based laser-produced plasmas. J. Appl. Phys..

[3] Hori T (2016). 100W Euv Light-Source Key Component Technology Update for Hvm.

[4] Yanagida T (2011). Characterization and Optimization of Tin Particle Mitigation and Euv Conversion Efficiency in a Laser Produced Plasma Euv Light Source.

[5] Kawasuji Y (2017). Key Components Technology Update of the 250W High-Power Lpp-Euv Light Source.

[6] Mizoguchi H (2016). Update of Euv source development status for Hvm lithography. J. Laser Micro Nanoeng..

[7] Gelderblom H (2016). Drop deformation by laser-pulse impact. J. Fluid Mech..

[8] Klein AL, Lohse D, Versluis M, Gelderblom H (2017). Apparatus to control and visualize the impact of a high-energy laser pulse on a liquid target. Rev. Sci. Instrum..

[9] Kurilovich D (2016). Plasma propulsion of a metallic microdroplet and its deformation upon laser impact. Phys. Rev. Appl..

[10] Sato Y (2017). Spatial profiles of electron density, electron temperature, average ionic charge, and Euv emission of laser-produced Sn plasmas for Euv lithography. Jpn. J. Appl. Phys..

[11] Sasaki A (2016). Modeling of Initial Interaction Between the Laser Pulse and Sn Droplet Target and Pre-Plasma Formation for the Lpp Euv Source.

[12] Schupp R, Torretti F, Meijer RA, Bayraktar M, Versolato OO (2019). Efficient generation of extreme ultraviolet light from Nd :Yag-driven microdroplet-tin plasma. Phys. Rev. Appl..

[13] Hudgins D, Gambino N, Rollinger B, Abhari R (2016). Neutral cluster debris dynamics in droplet-based laser-produced plasma sources. J. Phys. D.

[14] Gambino N, Brandstätter M, Rollinger B, Abhari R (2014). A hemispherical langmuir probe array detector for angular resolved measurements on droplet-based laser-produced plasmas. Rev. Sci. Instrum..

[15] Poirier L (2022). Strongly anisotropic ion emission in the expansion of Nd:Yag-Laser-produced plasma. Phys. Plasmas..

[16] Murakami M, Kang YG, Nishihara K, Fujioka S, Nishimura H (2005). Ion energy spectrum of expanding laser-plasma with limited mass. Phys. Plasmas..

[17] Hoffman J, Moscicki T, Szymanski Z (2011). The effect of laser wavelength on heating of ablated carbon plume. Appl. Phys. A.

[18] Moscicki T, Hoffman J, Szymanski Z (2013). The effect of laser wavelength on laser-induced carbon plasma. J. Appl. Phys..

[19] Galasso G, Kaltenbacher M, Tomaselli A, Scarpa D (2015). A unified model to determine the energy partitioning between target and plasma in nanosecond laser ablation of silicon. J. Appl. Phys..

[20] Wang Y, Hahn DW (2019). A simple finite element model to study the effect of plasma plume expansion on the nanosecond pulsed laser ablation of aluminum. Appl. Phys. A.

[21] Su MG (2017). Evolution analysis of Euv radiation from laser-produced tin plasmas based on a radiation hydrodynamics model. Sci. Rep.-UK.

[22] Aggoune S, Vidal F, Amara EH (2010). Numerical study of the expansion of metallic vapor plasma by a nanosecond laser pulse. Appl. Phys. A.

[23] Min Q (2016). Radiation properties and hydrodynamics evolution of highly charged ions in laser-produced silicon plasma. Opt. Lett..

[24] Stapleton MW, McKiernan AP, Mosnier JP (2005). Expansion dynamics and equilibrium conditions in a laser ablation plume of lithium: Modeling and experiment. J. Appl. Phys..

[25] Brandstätter M, Gambino N, Abhari RS (2018). Temporally and spatially resolved ion dynamics of droplet-based laser-produced tin plasmas in lateral expansion direction. J. Appl. Phys..

[26] Torrisi L (2006). Carbon-plasma produced in vacuum by 532 Nm–3 Ns laser pulses ablation. Appl. Surf. Sci..

[27] Zeng X, Mao XL, Greif R, Russo RE (2005). Experimental investigation of ablation efficiency and plasma expansion during femtosecond and nanosecond laser ablation of silicon. Appl. Phys. A.

[28] Vogt U (2001). Scaling-Up a Liquid Water Jet Laser Plasma Source to High Average Power for Extreme-Ultraviolet Lithography.

[29] Klein AL (2015). Drop shaping by laser-pulse impact. Phys. Rev. Appl..

[30] De Giacomo A, Dell'Aglio M, Gaudiuso R, Amoruso S, De Pascale O (2012). Effects of the background environment on formation, evolution and emission spectra of laser-induced plasmas. Spectrochim. Acta Part B.

[31] Shabanov SV, Gornushkin IB (2014). Two-dimensional axisymmetric models of laser induced plasmas relevant to laser induced breakdown spectroscopy. Spectrochim. Acta Part B.

[32] Kurilovich D (2018). Power-law scaling of plasma pressure on laser-ablated tin microdroplets. Phys. Plasmas.

[33] Murphy AB (2010). The effects of metal vapour in arc welding. J. Phys. D.

[34] Abdo Y, Rohani V, Fulcheri L (2017). An optimal method for the computation of the parameter Rs of the net emission coefficient approximation approach for determining the electrical and thermal characteristics of plasma arcs. J. Phys. D.

[35] Gleizes A, Gonzalez JJ, Liani B, Raynal G (1993). Calculation of net emission coefficient of thermal plasmas in mixtures of gas with metallic vapour. J. Phys. D.

[36] Sharma AK, Thareja RK (2005). Plume dynamics of laser-produced aluminum plasma in ambient nitrogen. Appl. Surf. Sci..

[37] Mahmood S, Rawat RS, Springham SV, Tan TL, Lee P (2010). Material ablation and plasma plume expansion study from Fe and graphite targets in Ar gas atmosphere. Appl. Phys. A.

[38] Sankar P, Shashikala HD, Philip R (2018). Ion dynamics of a laser produced aluminium plasma at different ambient pressures. Appl. Phys. A..

[39] Barthélemy O (2005). Influence of the laser parameters on the space and time characteristics of an aluminum laser-induced plasma. Spectrochim. Acta Part B.

[40] Cao SQ (2018). Dynamics and density distribution of laser-produced plasma using optical interferometry. Phys. Plasmas.

[41] Sai Shiva S (2019). Role of laser absorption and equation-of-state models on ns laser induced ablative plasma and shockwave dynamics in ambient air: Numerical and experimental investigations. Phys. Plasmas.

[42] Qiu R (2021). Mesoscopic kinetic approach for studying nonequilibrium hydrodynamic and thermodynamic effects of shock wave, contact discontinuity, and rarefaction wave in the unsteady shock tube. Phys. Rev. E..

[43] Zhang Z, Gogos G (2004). Theory of shock wave propagation during laser ablation. Phys. Rev. B.

